# Playing Video Games While Using or Feeling the Effects of Substances: Associations with Substance Use Problems

**DOI:** 10.3390/ijerph8103979

**Published:** 2011-10-18

**Authors:** Geoffrey L. Ream, Luther C. Elliott, Eloise Dunlap

**Affiliations:** 1 School of Social Work, Adelphi University, 1 South Avenue, Garden City, NY 11530, USA; 2 Institute for Special Populations Research, National Development and Research Institutes, 8th Floor, 71 West 23rd Street, New York, NY 10010, USA; E-Mails: luther@nyu.edu (L.C.E.); dunlap@ndri.org (E.D.)

**Keywords:** video games, caffeine, tobacco, alcohol, marijuana, addiction, dependence

## Abstract

This study tested the hypothesis that playing video games while using or feeling the effects of a substance—referred to herein as “concurrent use”—is related to substance use problems after controlling for substance use frequency, video gaming as an enthusiastic hobby, and demographic factors. Data were drawn from a nationally representative online survey of adult video gamers conducted by Knowledge Networks, valid n = 2,885. Problem video game playing behavior was operationalized using Tejeiro Salguero and Bersabé Morán’s 2002 problem video game play (PVP) measure, and measures for substance use problems were taken from the National Survey of Drug Use and Health (NSDUH). Separate structural equation modeling analyses were conducted for users of caffeine, tobacco, alcohol, and marijuana. In all four models, concurrent use was directly associated with substance use problems, but not with PVP. Video gaming as an enthusiastic hobby was associated with substance use problems via two indirect paths: through PVP for all substances, and through concurrent use for caffeine, tobacco, and alcohol only. Results illustrate the potential for “drug interaction” between self-reinforcing behaviors and addictive substances, with implications for the development of problem use.

## 1. Introduction

Between self-reinforcing behaviors and substances, there may exist a “drug interaction” which exacerbates addictive patterns. Addictive substances stimulate the brain’s natural reward pathways [1], and behavioral addictions involve the endogenous cannabinoid and related brain systems [2]. Dopamine plays a role in the self-reinforcing nature of substance use [3–5], and elevated dopamine plays a “gain-amplifying role” [6] in responses to “rewarding” Pavlovian stimuli [7]. As would be expected based on these shared biological mechanisms, survey studies find relationships between substance use problems and self-reinforcing behaviors like eating, gambling, internet use, sex, exercise, "workaholism," shopping, television viewing, and video games [8–13].

Such a “drug interaction” between self-reinforcing behaviors and addictive substances has been discovered in survey and laboratory findings that alcohol exacerbates problem gambling [14–18]. Part of the explanation offered for this is that alcohol contributes to disinhibition and risky decision making [19]. If this “drug interaction” effect is not, however, unique to the specific context of the alcohol/gambling combination but part of a larger pattern in which behavioral and substance addictions contribute to each other, then it should be evident in other substance/behavior combinations. It should also hold that engaging in self-reinforcing behavior while using or feeling the effects of a substance is uniquely associated with not only behavioral addiction, but substance use problems as well. In the present study, we focus on video game play. Video gaming is both similar to and distinct from gambling [20] in its self-reinforcing features [21] and degrees of prevalence and social acceptability, which all contribute to its unique characteristics as an addictive behavior [13].

The premise that video game playing is a potentially addictive behavior has empirical support [22]. Although the idea of video game addiction is not universally accepted [23], particularly as a discrete diagnostic category [24], and is not apparently likely to be included in the forthcoming DSM-V [25], problem video gaming behavior remains an area of scholarly and clinical [26,27] concern. A report by the American Medical Association’s Council on Science and Public Health called for more research on it [28]. A growing body of research on problem video gaming behaviors has emerged from within work on problem gambling [29] and adapted its measures [27], which originally came from Diagnostic and Statistical Manual (DSM) substance abuse/dependence criteria [30,31]. Prevalence estimates of significant problem video gaming behavior hover between 4.9% and 9% among video gamers internationally [11,32–35], with smaller numbers fitting stricter criteria for dependence [22,36].

Video game addiction has identifiable biological dimensions, which are similar to those for other behavioral addictions as well as substance dependence. An electroencephalography study found differences in processing of game-related cues between “casual” and “excessive” video game players suggestive of an addiction-like process among the “excessive” players [37]. Video game play stimulates dopamine [38] through the “reward” structures programmed into video games [39,40]. Dopaminergic medications indicated for ADHD and substance dependence help remediate problem video gaming behavior [41,42], and video games are associated with development of attention problems in children [43,44].

In addition to shared biological mechanisms, problem video gaming behavior and substance use have several risk correlates in common. Examples include mood disorders [33,45,46], impulsivity [33,47], attention deficit hyperactivity disorder symptoms [42,48–52], low social competence [53,54], low academic performance [55–58], and (in the case of violent video games) violence [59,60]. Although many of these studies on negative effects of problem video gaming behavior focus on children and youth [24], there are similar findings on adults [22], which implies that it would not be accurate to frame video game addiction as a youth problem behavior. Additional studies on adults would help distinguish problem video gaming behavior as an issue with addiction, rather than something parsimoniously explained by youth problem behavior theory [61].

Given shared biological mechanisms and risk correlates of problem video gaming behavior and substance use problems, as well as the general trend for behavioral and substance addictions to co-occur [13], this study investigated unique potential of playing video games while using or feeling the effects of a substance—referred to herein as “concurrent use”—to contribute to problem video gaming behavior and substance use problems. Its hypothesis was that concurrent use would explain variance in problem video gaming behavior and substance use problems that would not be explained by substance use frequency, video gaming as an enthusiastic hobby (video game play frequency, enjoyment, and consumer involvement), or demographics, including gender [30,34,62,63], race [64], age, and socioeconomic indicators. Analyses tested this hypothesis with respect to caffeine, tobacco, alcohol, and marijuana.

## 2. Methods

### 2.1. Participants and Recruitment

Participants were a subset of a nationally representative KnowledgePanel^®^ maintained by Knowledge Networks, a commercial online survey service provider. Knowledge Networks selects panel members via random-digit dial and address-based sampling methods, provides panel members with computers and internet access if needed, establishes informed consent, and collects basic demographic information. Once in the panel, members are randomly recruited via e-mail for client surveys, including this study. For each survey, participants receive “points” toward cash and other incentives offered by Knowledge Networks. For some client surveys, panel members are presented with screening questions and only allowed to participate if they meet specific criteria. For this survey, 15,642 e-mails were sent to panel members age 18 and over, and 9,215 (59%) completed the screening instrument. The valid sample was 42% female, 69% white, 11% Black, 13% Latino, 4% Asian, 2% Native (includes American Indian, Alaska Native, Native Hawaiian, and Pacific Islander), and 2% multiracial or other. More than half (58%) were currently either employed for wages or self-employed. The average participant was 40.4 years old (*SD* = 15.7), with annual income between $35,000 and $39,999, and had some college education but no degree.

The screener asked whether participants “regularly,” “occasionally,” or “never” participated in 11 different hobby activities in the past year, including video games. Participants who responded “regularly” or “occasionally” about video games were then asked how many hours of video and/or computer games they played in the past seven days. Participants who reported one or more hours, n = 3,380 (37%), were allowed to take the survey. Screening and the survey itself were conducted in English and Spanish. The Spanish version was professionally translated by Knowledge Networks using multiple translators and back-translation. It was also reviewed by the first author, who is fluent in written Spanish. Completion of the measure took an average of 10 minutes, which was the maximum median length feasible given constraints of the method and budget. The protocol for this study was reviewed and approved by all investigators’ Institutional Review Boards.

### 2.2. Measures

#### Substance use frequency

These measures were adapted from the National Survey of Drug Use and Health. (NSDUH, [65]). Participants chose, from a list, those substances they had used in the past 30 days. For each they chose, they were asked on how many of the past 30 days they had used it.

#### Substance use problems

NSDUH-based [65] abuse and dependence symptom items were presented for each substance used in the past 30 days. A set of questions was adapted for caffeine by using the full set of abuse/dependence items and leaving out those inapplicable to it (e.g., spending a lot of time obtaining/using/recovering from it, neglecting work and social life in order to use it). Because the full-length measures could not realistically be included in a survey constrained to a median length of 10 minutes, subsets were selected based on factor analyses of another dataset collected in this project (not yet published) which did include full-length measures—seven dichotomous items for caffeine, 11 Likert-scale items for tobacco, and 14 dichotomous items for each of alcohol and marijuana. For sets of dichotomous items, tetrachoric correlation matrices were factor analyzed. The goal of item selection was not to reproduce DSM-IV diagnoses but to measure problem substance use as a matter of degrees as authentically as possible within time constraints. Items were chosen that were highly correlated with the first/only factor, but not redundant—when collinearity resulted from everyone who reported one symptom also having reported another symptom, the less-frequently-reported symptom was left out. The final 5-item measure for caffeine included symptoms of tolerance, difficulty controlling use, desire to quit or cut down, withdrawal, and disregarding negative emotional or physical health consequences of use, CFI from confirmatory factor analysis = 0.962. Final measures for alcohol (CFI = 0.997) and marijuana (CFI = 0.998) included these plus neglecting positive activities and spending a lot of time obtaining or using, a total of 7 items each. The final measure for tobacco included four Likert-scale items for symptoms presently experienced including withdrawal, craving, worry over running out, and tolerance, CFI = 0.990.

#### Concurrent use with video games

For each substance that participants reported having used in the past 30 days, they were asked, “During the past 30 days, have you played video games while using (substance in question) or feeling its effects?”

#### Video game use and enjoyment

Participants were asked to list, via text entry, up to five video game titles they had “spent a lot of time playing in the past 12 months.” They were asked a series of questions about each title, including on how many days of the past 30 they had played it and how much they enjoyed it. Enjoyment was a single 7-point Likert scale in which 1 indicated “it was the worst game I’ve ever played,” four indicated “about the same as most games,” and 7 indicated “it was my single all-time favorite.” Because all dependent variables in the present analyses were person-level, within-person means were calculated to reflect the properties of the average game that person played. Entries for use and enjoyment variables were only considered valid for 2,885 participants who entered at least one valid title, and titles were considered valid if they could be uniquely identified in GameFaqs [66], a large and comprehensive database of user-generated content maintained and edited by an industry group.

#### Consumer involvement in video games

Another indicator of video game playing as an enthusiastic hobby [27,67–69] as distinct from addiction [70] was a measure of consumer involvement adapted from leisure and marketing studies [71–73]. It addresses attraction, centrality/importance, and self-expression, Cronbach’s α= 0.70.

#### Problem video game playing (PVP)

Because there is still debate about the definition of video game abuse/dependence [23,74,75], and symptoms of any disorder may constitute a problem in living even if they do not meet a clinical threshold, this study operationalized problem video gaming behavior with a continuous Likert scale measure (PVP, [31]). Like our measures for substance use problems, this scale also had to be abridged to fit into the 10-minute median time limit for this survey. Items were selected based on factor analysis of the same data we used to derive the substance use measures. For that study, the original 9-item Likert scale PVP measure had been slightly edited by splitting the longest item (“I have tried to control, cut back, or stop playing, or I usually play with the video games over a longer period than I intended”) into two separate items and deleting “with the” to produce a measure with a total of 10 items. The five highest loading items on the first/only factor across all four estimation procedures available in STATA 11.0 (principal factor, principal-component factor, iterated principal factor, and maximum likelihood factor) addressed increasing time spent playing (tolerance), difficulty controlling time spent playing, restlessness/irritability when can’t play (withdrawal), play to relieve negative affect (self-medication), and engaging in problem behavior in order to play games, CFI = 0.959, α = 0.74. Response choices ranged from “not at all true” to “extremely true,” so that participants who scored anywhere above the lowest possible score at least slightly positively identified with at least one item.

#### Demographics

Age, race/ethnicity, gender, education, income, and employment status were taken from the Knowledge Networks’ basic demographic survey. No data were missing on these variables. Income was categorized into 19 increments beginning with “less than $5,000” that are increasingly larger further up the scale until the final category, “$175,000 or more.” Education was an ordinal variable with 14 possible categories ranging from “no formal education” to “professional or doctoral degree.” Employment, for purposes of these analyses, was collapsed into categories of (1) working, either for wages or self-employed, or (2) non-working for any reason, e.g., disability, retirement, layoff, *etc*.

### 2.3. Approach to Analyses

All analyses employed post-stratification weights provided by Knowledge Networks so that estimates approximate what would have been obtained from a true random sample of English- and Spanish-speaking American adult video game players [76]. Calculated based on current data from the U.S. Census Current Population Survey, Knowledge Networks’ weights adjust for survey non-response and client surveys’ own sample designs, such as our screening procedure. We only present weighted estimates in the results section.

Bivariate relationships among study variables were computed using scale values for consumer involvement, PVP, and tobacco dependence, and count variables for the number of abuse/dependence symptoms for caffeine, alcohol, and marijuana. For these tables, significance flags were adjusted relative to convention in light of multiple tests, so that * indicates p < 0.01 rather than p < 0.05, ** indicates p < 0.001 rather than p < 0.01, *etc*.

For main hypothesis tests, structural equation models were run in MPlus 6.0, separately by substance. In each model, PVP and substance use problems were continuous latent variables measured by their observed components and allowed to correlate. MPlus is capable of creating a continuous latent variable from binary observed indicators, and this was done for the five indicators of caffeine use problems and the seven indicators of each of marijuana and alcohol use problems. PVP and substance use problems were regressed over the concurrent use binary variable, the observed indicator of substance use frequency, and a latent indicator of video gaming as an enthusiastic hobby. In a separate statement, concurrent use was regressed over substance use frequency and the latent variable for video gaming as an enthusiastic hobby. Substance use frequency and the latent variable for video gaming as an enthusiastic hobby were allowed to correlate. The latent variable of video gaming as an enthusiastic hobby was measured by the observed indicators of game enjoyment, hours played, and the scale score for consumer involvement. In the last statement specifying the model, all of these variables were regressed over demographic variables, so that the structural models described below refer to effects after demographic controls. The binary variable for concurrent use was accommodated in a mediating role through theta parameterization, and post-stratification weights were accommodated using means and variance adjusted weighted least squares (WLSMV) estimation.

**Figure 1:**
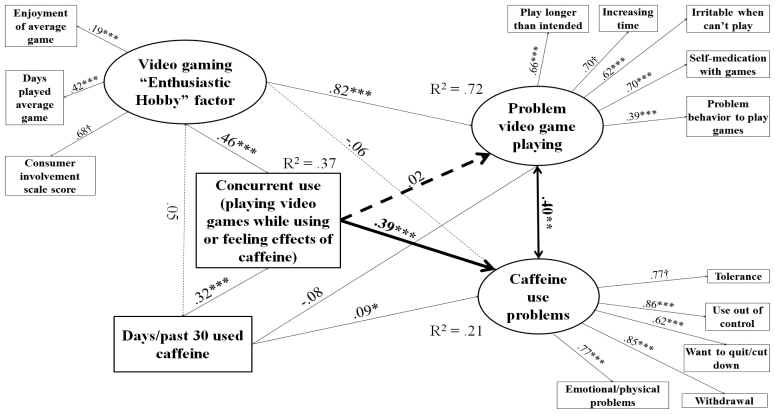
Structural equations model for effect of concurrent use on PVP and caffeine use problems among caffeine users. N = 1,961. Root mean square error of approximation (RMSEA) = 0.035, Comparative fit index (CFI) = 0.893. Tucker-Lewis index (TLI) = 0.850. Heavy lines indicate paths testing study hypotheses, light lines indicate control/measurement model paths. Solid lines indicate significant paths, and dashed lines indicate non-significant paths. Coefficients standardized after estimation; † indicates parameter constrained to be 1 for estimation. Model controls for age, educational level, gender, income, employment status, and race. * p < 0.05, ** p < 0.01, *** p < 0.001.

**Figure 2:**
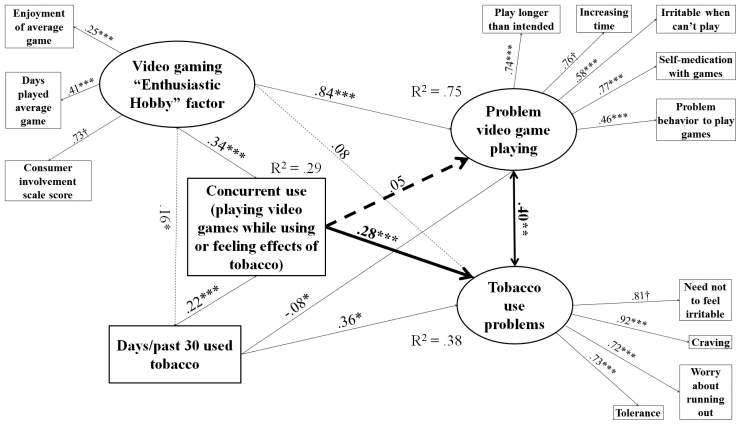
Structural equations model for effect of concurrent use on PVP and tobacco use problems among tobacco users. N = 683. Root mean square error of approximation (RMSEA) = 0.026, Comparative fit index (CFI) = 0.934. Tucker-Lewis index (TLI) = 0.904. Heavy lines indicate paths testing study hypotheses, light lines indicate control/measurement model paths. Solid lines indicate significant paths, and dashed lines indicate non-significant paths. Coefficients standardized after estimation; † indicates parameter constrained to be 1 for estimation. Model controls for age, educational level, gender, income, employment status, and race. * p < 0.05, ** p < 0.01, *** p < 0.001.

**Figure 3:**
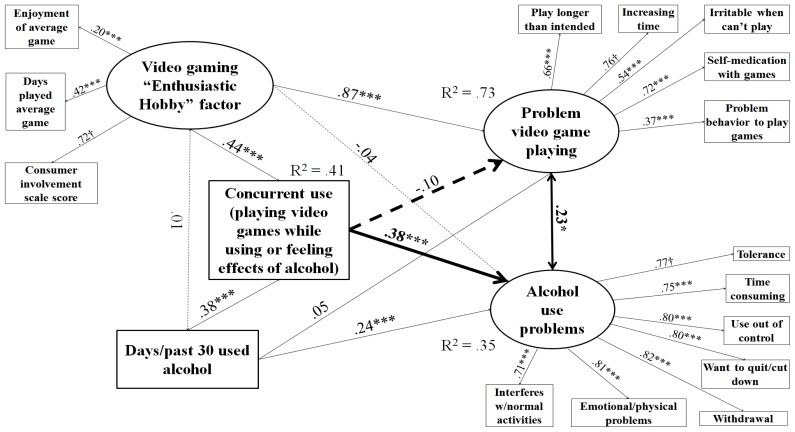
Structural equations model for effect of concurrent use on PVP and alcohol use problems among alcohol users. N = 1,018. Root mean square error of approximation (RMSEA) = 0.025, Comparative fit index (CFI) = 0.936. Tucker-Lewis index (TLI) = 0.915. Heavy lines indicate paths testing study hypotheses, light lines indicate control/measurement model paths. Solid lines indicate significant paths, and dashed lines indicate non-significant paths. Coefficients standardized after estimation; † indicates parameter constrained to be 1 for estimation. Model controls for age, educational level, gender, income, employment status, and race. * p < 0.05, ** p < 0.01, *** p < 0.001.

**Figure 4:**
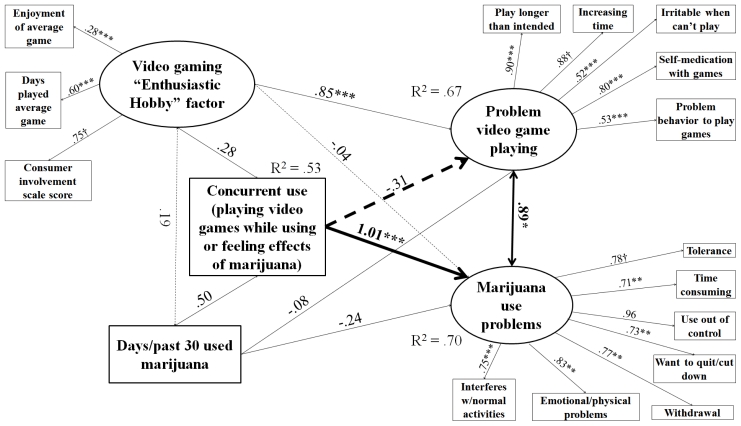
Structural equations model for effect of concurrent use on PVP and marijuana use problems among marijuana users. N = 133. Root mean square error of approximation (RMSEA) = 0.038, Comparative fit index (CFI) = 0.891. Tucker-Lewis index (TLI) = 0.857. Heavy lines indicate paths testing study hypotheses, light lines indicate control/measurement model paths. Solid lines indicate significant paths, and dashed lines indicate non-significant paths. Coefficients standardized after estimation; † indicates parameter constrained to be 1 for estimation. Model controls for age, educational level, gender, income, employment status, and race. * p < 0.05, ** p < 0.01, *** p < 0.001.

Models were determined to have acceptable fit based on RMSEA < 0.05, even though the lowest CFI was 0.89 and the highest was 0.94, which are just below the usual strict standard of 0.95 [77,78]. Alternative specifications were attempted that included only either the observed indicator for consumer involvement or video game play frequency in place of the “enthusiastic hobby” factor, and left “problem behavior to play games” out of PVP. These yielded CFI ≥ 0.95 for all four models and significant paths between concurrent use and PVP for caffeine, tobacco, and marijuana users. However, this specification would have limited the conceptual scope of the study. Observed components of the enthusiastic hobby factor were selected for conceptual completeness according to the logic of a formative indicator [79]; they were not expected to be redundant. Including all components of the enthusiastic hobby indicator was necessary in order to fully distinguish problem video gaming behavior from mere engagement [27]. Without them, we could not be sure the significant paths between concurrent use and PVP were not type I error due to underspecification. Another choice made for conceptual completeness was to leave the PVP measure intact despite the low-loading item and not reduce it even further relative to the original. Given that all RMSEAs were still < 0.05 [77,78] and some diminishment of CFI is forgivable when including variables that are not expected to be correlated but still need to be in the model for conceptual reasons [80], we determined the models described in Figures 1–4 to be the most authentic representation of the findings among the possibilities.

## 3. Results

Table 1 provides descriptive statistics on video game and substance use variables, as well as differences by categorical demographic factors.

Concurrent use with all substances was prevalent. Males reported higher consumer involvement, but females report higher enjoyment, and males (within this video gamers only sample) exhibited only marginally higher PVP. Females had more frequent use of and problems with tobacco. Males were, however, more frequent concurrent users with caffeine and alcohol than females. Although Blacks, Asians, and Native Americans had higher PVP than whites, Blacks had higher enjoyment and consumer involvement but not frequency, Asians had higher frequency but not enjoyment or consumer involvement, and Native Americans had higher consumer involvement but not frequency or enjoyment. Whites, however, were the only group to exhibit clearly higher rates of concurrent use issues, and then only with caffeine. The only demographic factor consistently associated with risk for problem use patterns was non-working status, and this only held for video games, tobacco, and alcohol.

Table 2 describes bivariate correlations among continuous study variables. Frequency of game playing, enjoyment of average game, consumer involvement, and PVP were all correlated with each other. All substance use problems variables were correlated with each other except for marijuana with tobacco and PVP. The positive correlation between days played and age was not, according to follow-up analyses (not shown), because of meaningful curvilinearity—the slope of the positive relationship between age and days played was steeper for younger participants and still positive, although more shallow, for older participants. Age was, however, negatively correlated with other game playing variables, including PVP. Consistent with findings about employment in Table 1, income and education were negatively correlated with video game playing frequency, consumer involvement, PVP, tobacco use frequency, tobacco use problems, and alcohol use problems.

Figures 1–4 describe results of path analysis models for caffeine, tobacco, alcohol, and marijuana users (respectively). For all models, the path from concurrent use to substance use problems was significant, and the correlation between PVP and substance use problems also was significant. Concurrent use was not directly associated with PVP. Video gaming as an enthusiastic hobby was indirectly associated with substance use problems through two paths. The first path was via PVP, which was significant for all four substances. The second was via concurrent use, which was only significant for caffeine, tobacco, and alcohol. An effect via concurrent use with marijuana may have been hard to distinguish because of the low sample size and the high rate of concurrent use among marijuana users (see Table 1). The pattern of significant paths described here also held if a dosage variable (e.g., number of caffeinated drinks, cigarettes, or alcoholic drinks per day of alcohol use) was used instead of the substance use frequency variable.

## 4. Discussion

Results confirmed that, in models accounting for shared variance between substance use problems and PVP [8,10] and controlling for frequency of substance use, video gaming as an enthusiastic hobby, and demographics, concurrent use (playing video games while using or feeling the effects of substances) was uniquely associated with substance use problems. They did not, however, confirm the hypothesized direct association between concurrent use and PVP. The same pattern of results occurred for all four substances studied. The lack of a significant direct association between concurrent use and PVP makes these results not wholly congruent with previous research findings that alcohol exacerbates problem gambling [14–19]. Rather, video gaming as an enthusiastic hobby emerged as a possible “third variable” associated with both concurrent use and PVP. Our models also distinguish the enthusiastic hobby factor as indirectly associated with substance use problems, through PVP (for all for substances) and concurrent use (for caffeine, alcohol, and tobacco). Although demographics are background variables in our models, our bivariate results for demographic variables echo earlier findings that socioeconomic stressors are just as relevant to problem video gaming behavior [64,81] as they are to substance addiction [82].

Factors contributing to this study’s validity are its nationally representative sample collected by a provider whose data are frequently used in research [83] and its use of sampling weights calculated by the provider to correct for biases and authentically represent the population under study [76]. The online nature of the sample and 59% response rate to the screening instrument probably cannot be argued to be limitations in and of themselves: Online data collection has demonstrated validity in this area [84] and, while new communications technology has diminished response rates to all survey methods (phone and postal mail included), point estimates remain stable across methods and at much lower response rates than ours [85]. Another strength of this study was its sample of adults, filling a need noted in previous work for more studies on adults [24] and ensuring that our findings cannot be parsimoniously explained by youth problem behavior theory [61].

In order to meet the 10-minute median length requirement for this survey, established measures for PVP [31] and substance use [65] had to be abridged. We ensured validity of our measures, in part, through use of structural equation modeling – had measures really been invalid, our models would not have had adequate overall fit. Further confidence in our substance use problems and problem video gaming behavior measures can be drawn from their significant bivariate correlations with each other, which are consistent with other research using these concepts [8,10,11]. Another limitation of our measures was that operationalization of our key construct of concurrent use depended on a single question: “During the past 30 days, have you played video games while using [substance in question] or feeling its effects?” Some participants may have interpreted it either too strictly (as an exclusive rather than inclusive “or”) or loosely (reporting on usual behavior rather than thinking specifically about the past 30 days). Although this variation in interpretation may have increased random error, it did not necessarily introduce bias. Further confidence in the validity of this measure can be drawn from the same pattern of associations involving it holding for all four substances under study. Other limitations of this study are those that it shares with every survey study, e.g., dependence on self-report data and participants’ recall of events 30 days or even a year ago. Finally, the scope of the implications of these findings is limited by their basis in cross-sectional data. Although our graphics include arrows indicating directions of effects, structural equation modeling in the context of a study like this mainly offers a heuristic for understanding associations among variables. Our results are not meant to support firm conclusions about causality or how these factors influence each other over time.

Even with these limitations, these findings contribute to a growing understanding that behavioral and substance addictions co-occur [8–13] and may contribute to each other through influencing or complementing each other’s actual use practices [14–18] and activating biologically mediated addictive processes [2–7]. Understanding concurrent engagement in self-reinforcing behavior and use of addictive substances may be an important consideration in addiction specificity [86], *i.e.*, variability in the development of co-occurring addictive patterns among those engaged in the prerequisite behaviors. Clinical implications of these findings, to the extent that they can be drawn from a non-clinical population, are that clients who present with behavioral addictions or substance use problems should be screened for both and also assessed for concurrent use.

## Figures and Tables

**Table 1:** Overall means/proportions for game playing and substance use variables, and differences by demographic factors.

		Overall mean*(sd)* or proportion	Gender		Race						Working	
		
			Male	Female	White	Black	Latino	Asian	Native	Other	Yes	No
**Video Games** weighted n(users) = 2,869.5	Days played average game	9.8(*8.5*)	9.5	10.2 +	9.9 (ref)	9.3	9.3	12.2 *	9.2	7.9	8.6	11.4 ***
	Enjoyment of average game	5.2(*1.1*)	5.19	5.30 *	5.21 (ref)	5.44 ***	5.24	5.14	5.30	5.12	5.22	5.26
	Consumer involvement	2.1(*0.8)*	2.23	2.03 ***	2.12 (ref)	2.38 ***	2.02 +	2.18	2.44 *	2.20	2.10	2.20 *
	Problem video game play	1.6(*0.6*)	1.58	1.53 +	1.53 (ref)	1.64 *	1.54	1.73 *	1.78 *	1.64	1.50	1.64 ***
		
**Caffeine** weighted n(users) = 1,849.3	Any use of caffeine	64%	63%	67% +	70% (ref)	44% ***	51% ***	63%	72%	66%	66%	62%
	Days/past 30 used caffeine a	24.4(*8.7*)	24.4	24.4	25.6 (ref)	19.6 ***	20.8 ***	22.0 **	21.5 *	23.2	24.6	24.1
	Caffeine use problems a	1.08(*1.40*)	1.01	1.18 *	1.07 (ref)	1.02	1.08	1.32	1.41	1.01	1.06	1.12
	Concurrent use with caffeine a	41%	45%	35% ***	44% (ref)	34% +	27% ***	19% **	35%	47%	39%	43%
		
**Tobacco** weighted n(users) = 748.7	Any use of tobacco	26%	27%	25%	27% (ref)	26%	19% *	19%	44% +	27%	23%	30% **
	Days/past 30 used tobacco a	24.0(*10.6*)	22.0	27.2 ***	24.4 (ref)	24.6	22.6	26.4	19.8 +	19.0 +	22.4	25.8 ***
	Tobacco use problems a	2.55(*1.20*)	2.37	2.83 ***	2.58 (ref)	2.51	2.32	3.19 +	2.44	2.08	2.36	2.76 ***
	Concurrent use with tobacco a	61%	62%	59%	61% (ref)	66%	49%	28% *	76%	89% +	52%	70% ***
		
**Alcohol** weighted n(users) = 964.6	Any use of alcohol	34%	40%	25% ***	35% (ref)	33%	31%	15% **	38%	41%	37%	29% ***
	Days/past 30 used alcohol a	11.0(*8.8*)	11.3	10.3	11.8 (ref)	9.0 *	8.4	8.0	8.2 **	12.6	10.4	12.0 *
	Alcohol use problems a	1.33(*1.70*)	1.34	1.33	1.28 (ref)	1.44	1.50	0.74	2.86 **	0.97	1.21	1.55 *
	Concurrent use with alcohol a	38%	42%	28% **	40% (ref)	42%	21%	14%	46%	38%	34%	44% *
		
**Marijuana** weighted n(users) = 162.0	Any use of marijuana	5.6%	6.4%	4.7%	5.7% (ref)	7.4%	5.5% **	0%	3.5%	7.5%	5.0%	6.6%
	Days/past 30 used marijuana a	19.1(*11.3*)	19.7	18.1	19.1 (ref)	17.3	21.1	b	b	16.9	19.5	18.8
	Marijuana use problems a	2.28(*2.10*)	2.50	1.86	2.07 (ref)	2.80	2.91	b	b	2.34	1.98	2.60
	Concurrent use with marijuana a	80%	84%	72%	80% (ref)	76%	77%	b	b	100%	78%	81%

a Only users of the substance in question included in these substance-specific analysis.

b Figures based on <5 real cases omitted.

+ p < 0.05,

* p < 0.01,

** p < 0.001,

*** p < 0.0001.

**Table 2:** Correlations among game playing, substance use, and continuous demographic variables.

	Days played	Game enjoyment	Consumer involvement	Problem play (PVP)	Caffeine days^a^	Caffeine problems^a^	Tobacco days^a^	Tobacco problems^a^	Alcohol days^a^	Alcohol problems^a^	Marijuana days^a^	Marijuana problems^a^
**Enjoyment of avg. game**	0.14 ***	1	0.18 ***	0.16 ***	0.02	0.07 *	0.05	0.08 +	−0.04	0.04	0.12	0.13
**Consumer involvement**	0.21 ***	0.18 ***	1	0.57 ***	−0.06 *	0.07 *	0.06	0.13 **	−0.05	0.14 ***	0.07	0.19 +
**Problem video game play**	0.28 ***	0.16 ***	0.57 ***	1	−0.08 **	0.24 ***	0.08 +	0.33 ***	−0.03	0.22 ***	−0.06	0.27 *
**Days caffeine use**	0.11 ***	0.02	−0.06 *	−0.08 **	1	0.09 **	0.14 *	0.08	0.16 ***	−0.06	0.44 ***	−0.10
**Caffeine use problems**	0.01	0.07 *	0.07 *	0.24 ***	0.09 **	1	0.02	0.33 ***	−0.12 *	0.37 ***	0.16	0.51 ***
**Days tobacco use**	0.17 ***	0.05	0.06	0.08 +	0.14 *	0.02	1	0.48 ***	0.11	−0.01	0.26 +	−0.21
**Tobacco use problems**	0.16 ***	0.08 +	0.13 **	0.33 ***	0.08	0.33 ***	0.48 ***	1	0.03	0.21 **	0.08	−0.11
**Days used alcohol**	0.08 +	−0.04	−0.05	−0.03	0.16 ***	−0.12 *	0.11	0.03	1	0.23 ***	0.16	−0.25 +
**Alcohol use problems**	0.02	0.04	0.14 ***	0.22 ***	−0.06	0.37 ***	−0.01	0.21 **	0.23 ***	1	0.01	0.31 *
**Days used marijuana**	0.18 +	0.12	0.07	−0.06	0.44 ***	0.16	0.26 +	0.08	0.16	0.01	1	0.24 *
**Marijuana use problems**	0.24 *	0.13	0.19 +	0.27 *	−0.10	0.51 ***	−0.21	−0.11	−0.25 +	0.31 *	0.24 *	1

**Age**	0.22 ***	−0.13 ***	−0.18 ***	−0.13 ***	0.24 ***	−0.16 ***	0.13 *	0.03	0.29 ***	−0.20***	0.11	−0.14
**Education**	−0.13 ***	−0.05 *	−0.07 **	−0.09 ***	0.00	0.00	−0.16 ***	−0.19 ***	0.03	−0.14 ***	−0.05	−0.13
**Income**	−0.11 ***	−0.05	−0.11 ***	−0.12 ***	0.06 *	−0.04	−0.13 **	−0.16 ***	0.00	−0.13 **	−0.18 +	−0.01

a Only users of the substance in question included in substance-specific analyses.

+ p < 0.05,

* p < 0.01,

** p < 0.001,

*** p < 0.0001.
